# The Effect of Motor Imagery Ability on Function and Proprioception in Myoelectric Prosthesis Users: Protocol for a Cross-Sectional Study

**DOI:** 10.2196/83787

**Published:** 2025-12-08

**Authors:** Ayşe Yazgan, Zeliha Candan Algun, Elif Aleyna Yazgan

**Affiliations:** 1 Department of Orthotics and Prosthetics Institute of Health Sciences Istanbul Medipol University Istanbul Turkey; 2 Orthopedic Prosthetics and Orthotics Program Vocational School of Health Services Istanbul Aydın University Istanbul Turkey; 3 Department of Physical Therapy and Rehabilitation and Orthotics and Prosthetics Faculty of Health Sciences Istanbul Medipol University Istanbul Turkey; 4 Department of Physiotherapy and Rehabilitation Faculty of Health Sciences Istanbul Nisantasi University Istanbul Turkey

**Keywords:** motor imagery, myoelectric prosthesis, amputation, motor function, proprioception

## Abstract

**Background:**

Motor imagery ability (MIA) is a critical cognitive process that enables the mental simulation of movements, facilitating skill acquisition in the use of myoelectric prostheses and their effective control in individuals who have undergone amputation. Although MIA is essential, its impact on upper extremity function and proprioception in myoelectric prosthesis users remains insufficiently studied.

**Objective:**

This study aims to evaluate MIA in individuals who have undergone transradial amputation and use myoelectric prostheses, compare it with a healthy control group, and examine its association with upper extremity functionality and proprioception.

**Methods:**

This cross-sectional study will include 15 individuals who have undergone transradial amputation (aged 18-65 y) and 15 age- and sex-matched healthy controls, recruited from prosthetics and orthotics centers in Istanbul, Turkey. Assessments will include MIA, hand mental rotation, upper extremity function, and proprioception. MIA will be measured using the Movement Imagery Questionnaire-3 and the Mental Chronometry Test. Hand mental rotation ability will be assessed via the Orientate mobile app. Upper extremity function will be evaluated using the Box and Block Test; Jebsen-Taylor Hand Function Test; and the Quick Disabilities of the Arm, Shoulder, and Hand questionnaire. Proprioception will be assessed through joint position sense measurements. All data will undergo appropriate statistical analyses.

**Results:**

Data collection began following funding from the Scientific and Technological Research Council of Turkey on April 25, 2025. As of November 2025, 8 participants with transradial amputation and 8 healthy controls have been recruited. Assessments are ongoing, and study completion is expected by June 2026.

**Conclusions:**

This study will evaluate MIA in individuals who have undergone transradial amputation and use myoelectric prostheses and explore its relationship with upper extremity function and proprioception. The findings are expected to guide the development of targeted rehabilitation strategies, particularly those incorporating motor imagery–based training for this population.

**International Registered Report Identifier (IRRID):**

DERR1-10.2196/83787

## Introduction

Amputation refers to the surgical removal of a limb along with its associated anatomical structures and is commonly performed due to trauma, infection, vascular diseases, or malignancies [1,2]. Among upper limb amputations, transradial amputations are the most common, accounting for more than 30% of the cases [3,4]. The anatomical complexity and functional importance of the forearm make transradial amputations particularly significant in both preoperative evaluation and postoperative management [5,6].

Following upper limb amputation, significant morphological and neurophysiological changes occur [7]. The loss of sensory input and visual feedback leads to neuroplastic remodeling within the central nervous system, resulting in extensive reorganization of cortical representation areas, particularly within the motor and somatosensory cortices [8,9]. These cortical topography alterations involve the reshaping of representational maps related to the missing limb and reflect both functional and structural adaptations [10,11]. Recent studies have highlighted that neuroplastic changes are especially pronounced in the somatosensory, premotor, and parietal cortices, which appear to form new neural connections despite the absence of peripheral sensory input following amputation [11,12]. Such neuroplastic adaptations are thought to play a crucial role in prosthesis use and motor learning.

Motor imagery (MI) is defined as the mental simulation of movement without actual physical execution and has gained considerable attention in neurodevelopment and rehabilitation research. During MI, brain regions involved in movement—such as the motor, premotor, and somatosensory cortices—are activated, highlighting their important role in motor planning and learning processes [13,14]. MI is typically classified into 2 main types: visual and kinesthetic imagery. Visual imagery involves mentally observing a movement from an external viewpoint, while kinesthetic imagery encompasses the internal experience of muscular activity and proprioceptive sensations [15]. Following amputation, neuroplastic changes in the somatosensory cortex may impair MI ability (MIA), reducing the capacity to mentally simulate movements [11]. Assessing MIA in individuals with limb loss is essential for understanding the clinical consequences of such neuroplastic alterations and tailoring personalized rehabilitation strategies. Deficits in MI can directly affect prosthetic adaptation and compromise the success of motor rehabilitation [16,17].

Myoelectric prosthetic systems function by detecting biopotential signals generated by residual muscle activity; however, effective prosthetic control relies not only on muscle contractions but also on cognitive processes, such as MI. Specifically, kinesthetic MI supports prosthetic control at the mental level, playing a key role in learning and adaptation. These cognitive processes are believed to promote neuroplasticity by enhancing the interaction between the central nervous system and muscular structures, thereby facilitating more efficient prosthesis control [18,19]. MI enables mental control of the prosthesis without direct muscle activation and improves cortical efficiency during prosthetic use. Consequently, both MI and proprioceptive awareness are fundamental for functional performance and neural adaptation in myoelectric prosthesis users [19].

In individuals who have undergone upper limb amputation, the preservation of MIA may significantly influence the process of adapting to myoelectric prostheses. Previous studies have demonstrated that individuals with higher MIA tend to perform functional tasks more effectively when using a prosthesis [20,21]. In this context, MI can be considered a potential facilitator for enhancing the functionality of myoelectric prostheses and improving upper limb performance. Furthermore, MI-based rehabilitation protocols have been shown to support neuroplasticity by strengthening the interaction between the central nervous system and prosthetic control mechanisms [19]. These findings underscore the clinical importance of assessing and enhancing MIA in myoelectric prosthesis users.

Proprioceptive feedback is a fundamental component in the perception and control of movement, as it transmits sensory information from muscles, tendons, and joints to the central nervous system [22]. Following amputation, the reduction or loss of these sensory inputs may impair MI processes, given that MI involves the mental simulation of both kinesthetic and somatosensory information [10,23]. Recent studies have shown that diminished proprioceptive input is associated with decreased MIA, which may lead to reduced performance in prosthetic control [24-26]. Therefore, preserving or restoring proprioceptive functions is crucial to supporting MI capacity in individuals with limb loss and, consequently, enhancing prosthesis use.

The physiological, psychological, and particularly cortical changes that occur following amputation require a multidimensional approach to managing individuals with limb loss. In this context, assessing MIA in people with transradial amputation has become increasingly important, as it contributes to the reorganization of motor control and supports cortical representation. Specifically, in the context of myoelectric prosthesis use, MI plays a complementary role by enhancing functional integration with the prosthesis and facilitating both movement planning and execution. This study aims to evaluate the MIAs of individuals with transradial amputation who use myoelectric prostheses by comparing them with healthy controls and investigate the impact of MI on functionality and proprioception. The findings are expected to provide a scientific foundation for integrating MI-based approaches into prosthetic rehabilitation programs.

## Methods

### Participants

The required sample size for the study was determined using G*Power software (version 3.1; Heinrich Heine University Düsseldorf). The calculation was based on the Movement Imagery Questionnaire-3 (MIQ-3) scores reported by Saimpont et al [27], which served as a reference for the primary outcome of this study.

Although Saimpont et al [27] reported a large effect size (Cohen *d*=1.41) in a healthy population, a more conservative estimate (Cohen *d*=0.8) was applied in this study’s power analysis to better reflect expected variability among participants who underwent transradial amputation. Using a 2-tailed independent samples *t* test, with an α of .05 and power (1–β) of .80, the required total sample size was calculated as 30 participants (n=15, 50% per group).

This sample size provides sufficient statistical power to detect large effects (Cohen *d*≥0.8) between groups while also allowing for potential participant dropouts. A sensitivity analysis showed that detecting moderate effects (Cohen *d*=0.6) would require approximately 18 participants to achieve equivalent power; however, as the study primarily aims to detect large between-group differences, the planned sample of 30 participants (n=15, 50% per group) was considered adequate.

Participants will be recruited from prosthetics and orthotics centers located in Istanbul, Turkey. The study group will consist of volunteers who meet the inclusion criteria; therefore, no probabilistic sampling method will be used. Inclusion criteria for the amputee and control groups are listed in Textbox 1.

**Inclusion criteria for the amputee and control groups.**

**Inclusion criteria for the amputee group**

- Aged between 18 and 65 years
- Completion of prosthetic rehabilitation and having used a prosthesis for at least 1 year
- Have a transradial level amputation
- Use of a 4-channel myoelectric-controlled transradial prosthesis
- Residual limb length between 5 and 25 cm
- A score of 3 or less on the visual analog scale for phantom or residual limb pain
- A score of 15 or less on the Beck Depression Inventory
- A score of 26 or greater on the Standardized Mini-Mental State Examination
- No diagnosis of any neurological, orthopedic, psychological (eg, schizophrenia and psychosis), or systemic disease
- Voluntary participation in the study

**Inclusion criteria for the control group**

- Aged between 18 and 65 years
- No diagnosis of any neurological, orthopedic, psychological, or systemic disease
- A score of 15 or less on the Beck Depression Inventory and 26 or greater on the Standardized Mini-Mental State Examination
- Voluntary participation in the study

Both groups will be included based on these criteria. No randomization will be applied between groups.

### Ethical Considerations

This study was approved by the noninterventional clinical research ethics committee of Istanbul Medipol University (E-10840098-202.3.02-2988) on May 9, 2024. All procedures will be conducted in accordance with the principles outlined in the Declaration of Helsinki. Before participation, all individuals will be informed about the study through both written and verbal explanations. Those who agree to take part will be asked to sign an informed consent form. The form explicitly states that participation is entirely voluntary and free of charge and that participants may withdraw from the study at any time without providing a reason or facing any consequences.

No additional costs will be incurred by the participants, and no compensation will be provided. All personal and identifying data will be kept strictly confidential. The collected data will be used solely for scientific purposes and without any commercial intent. Where necessary, anonymized data may be subjected to statistical analysis without the need for further ethics approval, provided that such use is covered by the initial consent.

### Study Design

This study is registered in the ClinicalTrials.gov database (NCT06541379) and uses a cross-sectional design with multicenter recruitment and single-center assessment. Participants will be recruited from several prosthetics and orthotics clinics located in Istanbul, Turkey, while all evaluations will be conducted at the prosthetics and orthotics research and application center of Istanbul Medipol University. All necessary institutional approvals were obtained before study initiation.

**Figure 1:**
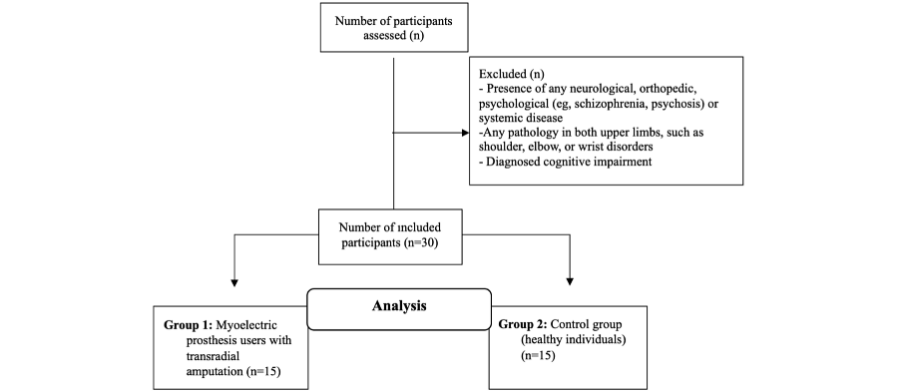
Study flowchart.

A total of 30 participants will be included: 15 (50%) individuals with unilateral transradial amputation who use a myoelectric prosthesis and 15 (50%) healthy control participants matched for age and sex. The study flowchart describing the recruitment and assessment process is presented in Figure 1.

The study timeline and procedures are summarized as follows: ethics approval was obtained from the institutional ethics committee on May 9, 2024, and funding was received from the Scientific and Technological Research Council of Turkey (TÜBİTAK) on April 25, 2025. Participant recruitment and data collection started on April 25, 2025, with 15 participants planned per group. Assessments and interventions include tests of MI ability, upper limb functional tests, and proprioception test. Follow-up will include ongoing monitoring and assessment of all participants. Data analysis will be completed after all data are collected. The study is expected to be completed in June 2026.

The sampling frame consists of all eligible individuals with unilateral transradial amputation who meet the inclusion criteria across the collaborating clinics during the recruitment period. Participants will be recruited consecutively, applying identical inclusion and exclusion criteria across centers.

Although participants will be recruited from different clinics, all assessments will be performed by a single trained researcher (the first author) using the same equipment and standardized procedures at the prosthetics and orthotics research and application center. This approach ensures measurement consistency and minimizes the risk of selection bias.

This study was reported in accordance with the STROBE (Strengthening the Reporting of Observational Studies in Epidemiology) statement for cross-sectional studies (Multimedia Appendix 1). The related TÜBİTAK 1002-A project report is provided in Multimedia Appendix 2.

### Outcome Measures

According to the study hypothesis, the primary outcome is MIA assessed using the MIQ-3, which was selected as the primary end point given its central role in prosthetic motor control, motor learning, and functional adaptation in individuals who have undergone transradial amputation.

The secondary outcomes include the following:

- Upper-limb functional performance, assessed using the Box and Block Test (BBT), Jebsen-Taylor Hand Function Test (JTHFT), and the Disabilities of the Arm, Shoulder, and Hand (QuickDASH) questionnaire
- MI performance indicators, including the Mental Chronometry Test (MCT) and hand mental rotation task, as secondary measures of MIA
- Proprioceptive and sensorimotor accuracy, measured by joint position sense (JPS) error using a validated digital goniometer app (DrGoniometer [CDM SrL])
- General balance and flexibility, evaluated by the Functional Reach Test (FRT) as an indicator of overall functional stability
- Cognitive and psychological screening, including the Standardized Mini-Mental State Examination (SMMSE), Beck Depression Inventory (BDI), and Visual Analog Scale (VAS) for pain intensity

Each participant will be assessed once, as this is a cross-sectional study with no intervention. To minimize potential bias, all assessments will be performed by trained evaluators following standardized protocols. Participants will be recruited according to predefined eligibility criteria to reduce selection bias, and data will be systematically recorded to prevent information bias. As no intervention is applied, performance bias is not applicable.

To minimize the risk of type I error resulting from multiple comparisons, the Bonferroni correction will be applied to secondary outcome analyses.

### Demographic Information

Demographic data will be collected through face-to-face interviews using a standardized data collection form developed by the research team. The recorded variables will include age, sex, height, weight, BMI, educational level, occupation, year and cause of amputation, dominant side, side of amputation, duration of prosthesis use, current medication use, and history of previously used prosthetic systems. For individuals with transradial amputation, stump length will be measured using a tape measure as the distance from the olecranon to the most distal end of the residual limb.

### SMMSE Description

The SMMSE is a brief 30-item test designed to assess cognitive functions, including attention, memory, orientation, calculation, language, and visuospatial skills. In this study, it will be used solely for participant eligibility screening. Individuals scoring 26 or higher will be included [28].

### BDI Description

The BDI is a 21-item self-report questionnaire used to measure an individual’s level of depression. Each item is rated on a scale from 0 to 3. In this study, only individuals with a score of 15 or lower will be included [29].

### VAS Description

The VAS is a measurement tool rated from 0 to 10 used to assess pain intensity. In this study, individuals who have undergone amputation with a VAS score of no more than 3 for phantom or residual limb pain will be included [30].

### MIQ-3 Description

The MIQ-3, originally developed by Williams et al [31], will be used to assess imagery ability in all individuals included in the amputee and control groups. The Turkish validity and reliability study of this questionnaire was conducted by Dilek et al [32]. The MIQ-3 consists of 12 items and evaluates internal visual imagery, external visual imagery, and kinesthetic imagery abilities through 4 actions for each domain. These actions include leg raising, jumping, shoulder flexion, and forward bending. The movements are designed to assess MI capacity in internal, external, and kinesthetic dimensions.

Participants will initially perform each movement physically. They will then be asked to mentally perform the same movements as a cognitive task. This mental imagery task will be rated using a 7-point Likert-type scale (1=very hard to see or feel and 7=very easy to see or feel). For scoring, the average score of the 4 movements corresponding to each subdimension will be calculated.

### MCT Description

MCT is an objective method used to evaluate the temporal accuracy of MI [33]. In this study, the MCT will be administered using the BBT. Participants will first be asked to physically transfer 15 blocks from one compartment to another, and the time taken will be recorded. Subsequently, the same task will be mentally imagined after removing the test apparatus from view. The duration for the imagined transfer of each block will be verbally indicated by the participant and recorded accordingly.

The difference between the actual and imagined durations will be used to calculate the mental chronometry ratio, using the following formula:

mental chronometry ratio = |actual time − mental time| / actual time

A ratio closer to 0 indicates that the imagined movement duration closely approximates the actual movement, reflecting a higher level of MIA.

### Orientate Mobile App Description

The Orientate app (Reflex Pain Management Ltd) is a rehabilitation tool focused on MI and laterality recognition (left and right discrimination). It is used to assess mental rotation ability. Participants will be presented with 25 hand images at various angles and asked to determine, as quickly as possible, whether each image depicts a left or right hand. The number of correct and incorrect responses, the number of skipped trials, and reaction times will be recorded [34].

### BBT Description

**Figure 2:**
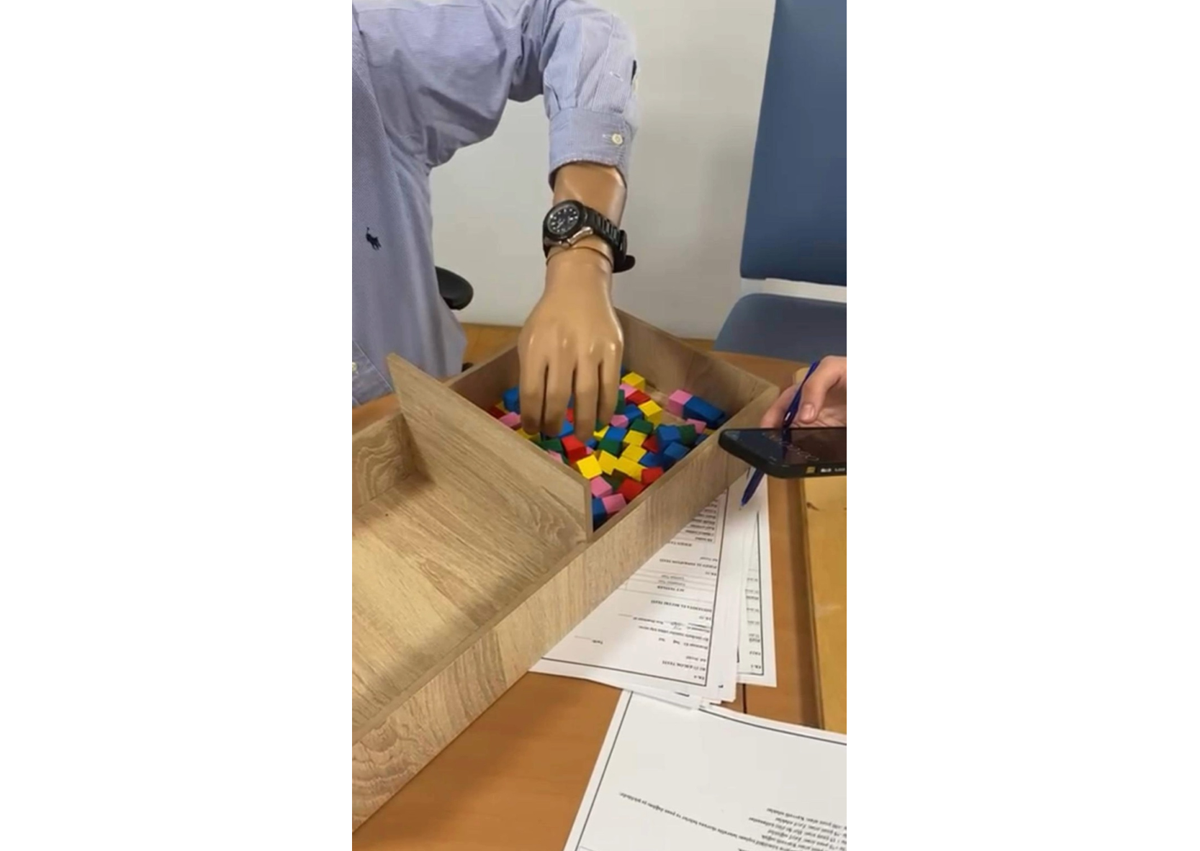
Box and Blocks Test in a participant who has undergone transradial amputation. The participant moves blocks between compartments within 60 seconds using the prosthetic hand.

The BBT is a widely used standardized assessment tool for evaluating gross motor skills and hand function. Originally developed by Mathiowetz et al [35], the test uses 150 wooden cubes, each measuring 2.5 cm, and a wooden box divided into 2 compartments. Participants are instructed to transfer as many cubes as possible, 1 at a time, from one compartment to the other within 60 seconds while seated at a table. The total number of cubes successfully transferred will be recorded as the test score (Figure 2).

### JTHFT Description

**Figure 3:**
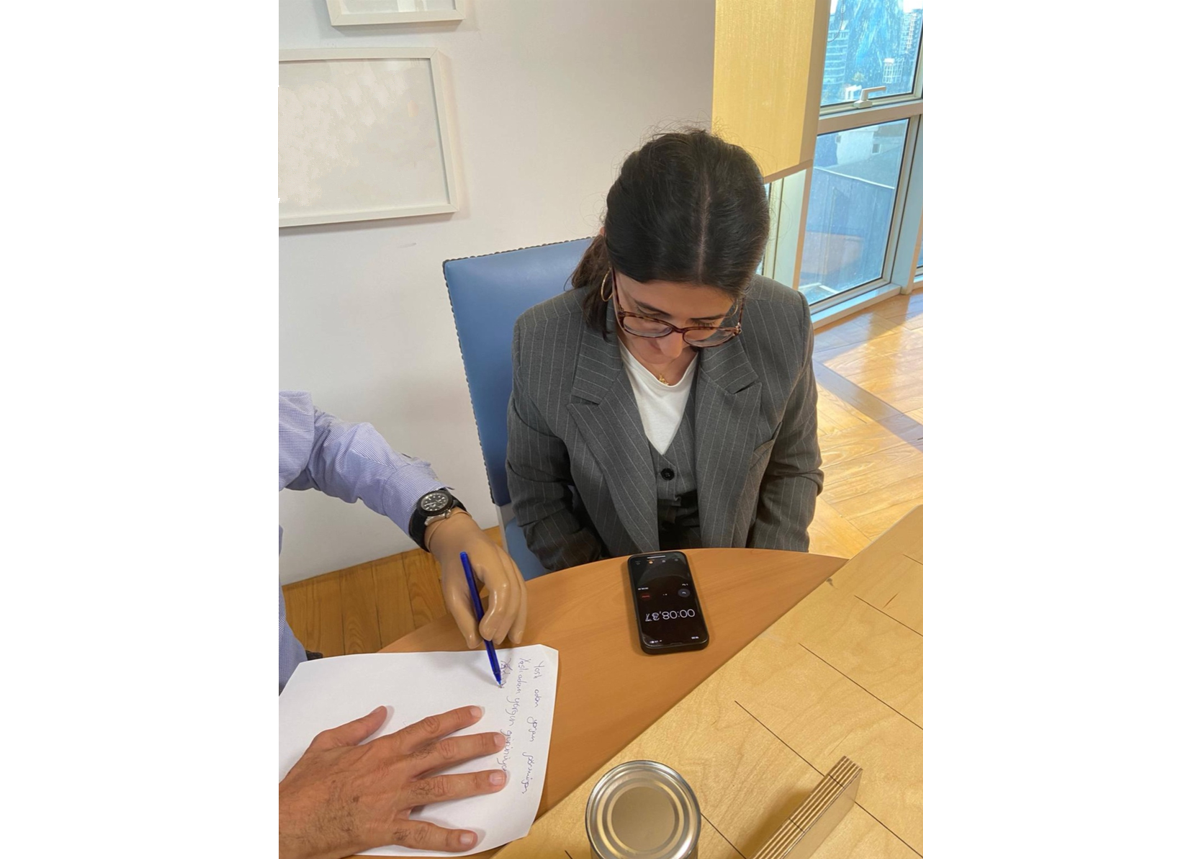
Jebsen-Taylor Hand Function Test performance in a participant who has undergone transradial amputation. The participant completes standardized hand tasks using the prosthetic hand.

The JTHFT, originally developed by Jebsen et al [36], will be used as a performance-based assessment to evaluate hand function in activities of daily living. The test will include various types of grasps and will assess the speed at which participants complete specific tasks. Standardized materials, which will be readily available in clinical settings, will be used for the test. Participants will be required to complete 7 distinct tasks: writing, turning over cards, picking up small objects, simulated feeding, stacking checkers, picking up large light objects, and picking up large heavy objects. Each task will be performed separately with both the dominant and nondominant hands. The test score will be calculated based on the time taken by participants to complete each task (Figure 3).

### FRT Description

In the FRT, the participant will stand on a flat surface, positioned sideways relative to the testing setup, with feet shoulder-width apart and stable. The test arm will be lifted laterally to shoulder height, with the fingers extended. Without moving their base of support, the participant will be instructed to reach forward as far as possible using only the upper extremity. The distance between the initial fingertip position and the furthest point reached during the movement will be measured in centimeters (cm) using a measuring tape. To enhance the reliability of the test, the measurement will typically be repeated 3 times, and the average of the 3 trials will be recorded [37]. Although FRT primarily assesses balance and reach, it provides additional information about reaching ability with the upper-limb prosthesis, which may indirectly reflect functional integration and MI performance.

### QuickDASH Questionnaire Description

The QuickDASH questionnaire will be used as a self-reported instrument designed to assess activity limitations and participation restrictions of the upper extremity [38]. The Turkish validity and reliability of the questionnaire were established by Düger et al [39]. The QuickDASH questionnaire consists of 11 items and aims to evaluate the level of difficulty individuals experience during daily living activities. Responses will be rated using a 5-point Likert scale (1=no difficulty, 2=mild difficulty, 3=moderate difficulty, 4=severe difficulty, and 5=unable to perform). Questionnaire scores will be recorded for both groups included in the study.

### JPS Description

To evaluate proprioceptive function relevant to prosthetic control, the JPS assessment will be used. JPS measures an individual’s ability to replicate a given joint angle and can be conducted using combinations of passive and active movements, under eyes-closed conditions [22].

Considering the amputation levels of the individuals who have undergone transradial amputation, all participants will undergo JPS assessment of the shoulder and elbow joints, performed bilaterally (dominant and nondominant sides where applicable) with standardized positioning while standing comfortably with feet shoulder-width apart and arms relaxed at the side before each trial. In the amputee group, assessments will be conducted with the prosthesis worn and the terminal device maintained in the closed position to ensure consistency across participants. For passive JPS, the investigator will move the limb to the target angle (eg, 30°) and then return it to the starting position, and the participant will estimate the joint angle without moving it. Participants’ vision will be occluded during assessment to prevent visual feedback, ensuring blinding to the target angle. For active JPS, the investigator first demonstrates the target angle with the participant’s eyes open. Subsequently, with eyes closed, participants actively replicate the joint angle without any physical guidance.

For the shoulder joint, angles of 90° abduction and 90° flexion will be used; and for the elbow joint, angles of 30°, 60°, and 90° flexion will be assessed. All joints will be positioned consistently at the same starting reference point for each trial to ensure measurement consistency across participants. Each angle will be measured 3 times, and the mean value will be recorded. Trials with outlier values exceeding 2 SDs from the mean or trials with measurement errors will be excluded.

All measurements will be conducted using the validated digital goniometer app DrGoniometer [40,41], which has demonstrated high validity and reliability for upper-limb joint measurements, including forearm supination and elbow flexion [40,41], although it has not been specifically validated in populations who have undergone amputation. Correctness (+5° and –5°) and reaction-time metrics are predefined. Absolute error, constant error, and variable error will be calculated to quantify accuracy and consistency. Due to the nature of the assessments, assessor blinding was not feasible; however, all measurements were conducted using a standardized protocol with fixed positioning and predefined verbal instructions to minimize bias

Covariates such as age, sex, amputation level, prosthesis experience, and tested side will be recorded and adjusted for in the statistical analyses.

All assessments will be completed within approximately 90 minutes, including short breaks as needed, in the following standardized order: MIQ-3, MCT, JTHFT, QuickDASH questionnaire, FRT, and finally JPS.

### Statistical Analysis

All analyses will be performed using SPSS (version 25.0; IBM Corp). Continuous variables will be checked for normality using visual inspection (histograms and Q-Q plots) and the Shapiro-Wilk test. Descriptive statistics will be reported as means and SDs for normally distributed variables or medians and IQRs for nonnormal variables. Categorical variables will be reported as counts and percentages. The details are presented in Textbox 2.

**Detailed statistical analysis plan.**

**Demographics**

- Amputee (group 1) and control (group 2) demographics will be compared. Normally distributed continuous variables will be assessed using an independent samples t test, nonnormal continuous variables will be assessed using the Mann-Whitney U test, and categorical variables will be assessed using the chi-square or Fisher exact test.

**Motor imagery (MI) variables**

- Measures will include the Movement Imagery Questionnaire-3, including its subscales: kinesthetic MI, internal MI, external MI, and total score as well as the Orientate app accuracy and completion time, and motor execution time–MI time for the dominant and nondominant hand.
- Comparisons will be performed using the analysis of covariance, adjusting for age, dominance, and residual limb length.
- If the analysis of covariance assumptions are not met, nonparametric alternatives will be applied.

**MI and upper limb function**

- Correlations between MI and functional tests (Box and Block Test; Jebsen-Taylor Hand Function Test subtests; Functional Reach Test; and Disabilities of the Arm, Shoulder, and Hand questionnaire) will be analyzed using Pearson or Spearman correlation, depending on normality.
- Regression analyses will examine the predictive effect of MI on function, adjusting for age, dominance, and residual limb length.

**MI and proprioception**

- Correlations between MI and proprioception measures (elbow 30°, 60°, and 90°, and shoulder 90° flexion and abduction) will be conducted.
- Regression models will evaluate predictors of proprioceptive performance, with covariates age, dominance, and residual limb length.

**Amputee-specific analyses**

- Dominant-side amputation will be coded as a binary variable (yes or no).
- Differences in MI, functional outcomes, and proprioception outcomes between participants with amputation of the dominant limb and participants with amputation of the nondominant limb will be analyzed using appropriate parametric or nonparametric tests.
- Associations with MI outcomes will be examined using correlation and regression analyses.

**Missing data, outliers, and multiple comparisons**

- Missing data will undergo listwise deletion.
- Trials with outlier values exceeding 2 SDs from the group mean will be excluded. Sensitivity analyses with and without outliers will be performed.
- The Bonferroni correction will be applied for multiple comparisons.

**Significance**

- All tests will be 2-tailed, with *P*<.05 considered statistically significant.

## Results

This study was approved by the noninterventional clinical research ethics committee of Istanbul Medipol University (E-10840098-202.3.02-2988) on May 9, 2024. Following funding from the TÜBİTAK on April 25, 2025, participant enrollment began. As of November 2025, a total of 16 participants have been recruited, including 8 (50%) individuals with transradial amputation and 8 (50%) healthy controls. All assessments for these participants have been completed, and the data have been entered into the study database. Preliminary data cleaning has started; however, statistical analyses have not yet been conducted. Recruitment and data analysis are ongoing, with study completion expected by June 2026.

## Discussion

### Expected Outcomes

This study protocol is designed to compare MIA between individuals who have undergone transradial amputation and use myoelectric prostheses and healthy individuals as well as evaluate the impact of this ability on upper limb functionality and proprioceptive sensation. By assessing MI capacity, upper extremity function, and proprioception together, this study aims to provide a multidimensional perspective on cognitive and sensorimotor processes in this population.

Existing literature indicates that a high level of MIA may positively influence functional performance and proprioceptive sensation in individuals who have undergone amputation, particularly enhancing functional gains during the preprosthetic and early prosthetic use phases [17,19]. Previous studies involving unilateral upper limb prosthesis users have shown that individuals who have undergone amputation can perform similarly to healthy individuals on hand mental rotation tasks, which are considered cognitive indicators of MIA [42,43]. These findings suggest that the reorganization of body schema following amputation could support MI processes. In line with this, McClanahan et al [44] demonstrated that individuals who have undergone transradial amputations and use myoelectric prostheses can achieve high levels of muscle coordination during prosthesis use, potentially preserving motor control strategies and contributing positively to MIA. With this background, our study is expected to contribute by evaluating the similarities and differences reported in the literature within our own sample, considering body schema reorganization, motor control strategies, and prosthesis use experience.

In previous research, both self-report and performance-based assessment tools, such as the MIQ-3, MCT, and Orientate app, have been shown to be effective in evaluating MIA [45]. For example, Martínez-Rolando et al [46] combined subjective visual and kinesthetic imagery ratings with performance-based measures of response time and accuracy. Similarly, Saruco et al [47] used the MCT to investigate temporal congruence between imagined and actual movement durations in individuals who have undergone lower limb amputation. These approaches demonstrate that MI can be evaluated not only in terms of vividness but also in temporal accuracy. In addition, mobile apps, such as Orientate, integrate imagery tasks with timing measurements, providing an objective assessment of both subjective and temporal parameters [48]. In alignment with this evidence, our study will incorporate these 3 parameters to obtain more comprehensive and objective data regarding the cognitive-motor capacity of individuals who have undergone amputation.

Ensuring that upper extremity function assessments are both reliable and clinically relevant is essential for prosthesis users. The literature identifies the BBT, the JTHFT, and the QuickDASH questionnaire as valid, reliable, and practical tools in clinical settings [49]. Our protocol includes these assessments to objectively measure motor performance and functionality. Furthermore, as shown by Gu et al [50], upper extremity motor skills may be closely related to neural activity during MI, highlighting the cognitive-motor link. Therefore, using a combination of QuickDASH, BBT, and JTHFT is expected to allow a comprehensive evaluation of both subjective and performance-based functional outcomes.

Proprioception, particularly JPS, plays a key role in effective motor control and may also influence MI processes. Clinical and digital JPS assessments, using both active and passive movements, have demonstrated accuracy and reliability [24,40]. In this protocol, proprioception will be evaluated using the DrGoniometer app, chosen for its validated accuracy and ability to perform digital angular measurements [51,52]. Shoulder abduction and flexion and elbow flexion at predefined angles will be assessed to ensure standardization. Previous studies suggest that proprioceptive feedback can enhance prosthetic control and support MI by aligning mentally simulated movement with sensory representations [26,53].

By integrating MI, upper limb function, and proprioception assessments, this study protocol aims to explore their interrelationships in a novel way. The expected outcomes include evidence to guide personalized rehabilitation approaches that combine cognitive and sensorimotor training.

### Strengths and Limitations

A key strength of this protocol is its multidimensional evaluation strategy, which combines subjective, performance-based, and digital assessment tools supported by literature. The inclusion of both healthy controls and myoelectric prosthesis users will allow for comparative analysis. However, this study is limited by its single-center design, specific prosthesis type (myoelectric only), and proprioception measurement restricted to predefined joint angles. In addition, sample size and recruitment feasibility may influence generalizability.

### Conclusions

Data collection and assessments are currently in progress, with data analysis planned after all participants have been assessed.

## Appendix

Multimedia Appendix 1 STROBE (Strengthening the Reporting of Observational Studies in Epidemiology) checklist for cross-sectional studies used in this study.

Multimedia Appendix 2 Peer-review report from the Tübitak 1002-A Rapid Support Module Review Committee, Scientific and Technological Research Council of Turkey.

## Abbreviations

BBT: Box and Block Test
BDI: Beck Depression Inventory
FRT: Functional Reach Test
JPS: joint position sense
JTHFT: Jebsen-Taylor Hand Function Test
MCT: Mental Chronometry Test
MI: motor imagery
MIA: motor imagery ability
MIQ-3: Movement Imagery Questionnaire-3
QuickDash: Disabilities of the Arm, Shoulder, and Hand
SMMSE: Standardized Mini-Mental State Examination
STROBE: Strengthening the Reporting of Observational Studies in Epidemiology
TÜBİTAK: Scientific and Technological Research Council of Turkey
VAS: visual analog scale

## Data Availability

The datasets generated or analyzed during this study are available from the corresponding author on reasonable request.
