# Supplementary material for: The Effect of Motor Imagery Ability on Function and Proprioception in Myoelectric Prosthesis Users: Protocol for a Cross-Sectional Study
Source: JMIR Res Protoc. 2025 Dec 8;14:e83787. doi: 10.2196/83787 (PMC12723360; doi:10.2196/83787)
Supplement: Multimedia Appendix 2 [file resprot_v14i1e83787_app2.pdf]

TÜBİTAK

T.C.
TÜRKİYE BİLİMSEL VE TEKNOLOJİK ARAŞTIRMA KURUMU BAŞKANLIĞI
Araştırma Destek Programları Başkanlığı

Sayı : B.14.2.TBT.0.06.03.02-161-532238 11/12/2024

Konu : 324S776 Numaralı Proje Karar Yazısı

Sayın Ayşe YAZGAN

"1002-A Hızlı Destek Modülü" kapsamında Kurumumuza sunmuş olduğunuz 324S776 numaralı ve "Myoelektrik Protez Kullanan Transradial Amputelerde Motor İmgeleme Yeteneğinin Üst Ekstremite Fonksiyonelliği Ve Propriyosepsiyon Üzerine Etkisinin Araştırılması" başlıklı projenize ilişkin bilimsel değerlendirme süreci tamamlanmıştır.

Desteklenmesine karar verilen proje önerinizin ilgili mevzuat çerçevesinde, mali ve benzeri konularda değerlendirme çalışmalarına başlanmıştır. Proje sözleşmenizin yapılabilmesi için gerekli olan belgelerin Panel Yönetim Sistemi (PYS) üzerinden ilgili Gruba iletilmesi gerekmektedir. Belgelerin iletiminin ardından projelere ait sözleşme ve diğer belgeler imzalanmak üzere tarafınıza gönderilecektir.

Başarınızı tebrik eder, saygılar sunarım.

Dr. Hatice Mahur TURAN
Sağlık Bilimleri Araştırma Destek
Grubu (SBAG)
Grup Koordinatörü V.

PUAN SEVİYESİ: A

A: Çok İyi B: İyi C: Orta D: İyi Değil E: Yetersiz F: Özgün Değeri Yetersiz

Panel puanı A ve B seviyesinde olan projeler desteklenmiştir.

* Bir kişi kariyer hayatı boyunca 1002-A Hızlı Destek Modülü ve 1002-B Acil Destek Modülü kapsamında toplamda en fazla beş kez proje yürütücüsü olarak görev alabilir.

TÜBİTAK

PROJE ÖNERİSİ BİLİMSEL DEĞERLENDİRME RAPORU

**PROJE NO**	**324S776**
**PROJE YÜRÜTÜCÜSÜ**	**Doktora Öğrencisi AYŞE YAZGAN**
**PROJE BAŞLIĞI**	**Myoelektrik Protez Kullanan Transradial Amputelerde Motor İmgeleme Yeteneğinin Üst Ekstremite Fonksiyonelliği Ve Propriyosepsiyon Üzerine Etkisinin Araştırılması**

1. ÖZGÜN DEĞER

Revize proje önerisi, myoelektrik protez kullanan transradial amputelerde motor imgeleme yeteneğini değerlendirmek ve motor imgeleme yeteneğinin, fonksiyonellik ve propriyosepsiyon duyusu üzerine olan etkisini araştırmak amacıyla planlanmıştır. Maddi hata olarak kabul edilen hipotezler bölümünde, hipotez sayısına ilişkin bir karmaşa olduğu dikkat çekmektedir. Literatürde amputasyon sonrası motor imgeleme yeteneğinin değerlendirildiği farklı çalışmalar bulunmaktadır. Bununla birlikte proje önerisinde myoelektrik protezi etkinleştirmek için gereken sinyallerin üretimi açısından motor imgelemenin önemi ve myoelektrik üst ekstremite protezi kullanan bireylerde motor imgeleme yeteneği ile ilgili literatürdeki eksiklik ortaya konmuştur. Bu nedenle proje önerisinin özgün değeri yüksek olarak değerlendirilmiştir.

2. YÖNTEM

Proje önerisinde uygulanacak yöntemler açık ve anlaşılır bir biçimde sunulmuştur. Gerekli literatüre atıf yapılmıştır. Yöntem öngörülen amaç ve hedeflere uygundur. Araştırma tasarımı, bağımlı ve bağımsız değişkenleri, istatistiksel yöntemleri açık ve tutarlı biçimde açıklanmıştır. Yapılan revizyon sonrası yöntemdeki eksiklikler tamamlanmıştır.

3. PROJE YÖNETİMİ

a. **Yönetim Düzeni:** Proje ekibi, görev dağılımı ve iş paketleri bakımından projeyi gerçekleştirmek için yeterli ve uygundur. Projenin başarı ölçütleri ölçülebilir ve izlenebilir olarak verilmiştir.

b. **Risk Yönetimi:** Projenin gerçekleşmesini etkileyebilecek riskler ve alınacak tedbirler yeterli biçimde ifade edilmiş, revizyon sonrası yapılan düzenlemelerle ilgili kısım geliştirilmiştir.

4. YAYGIN ETKİ

Proje önerisinden beklenen yaygın etkiler açık olarak ifade edilmiştir. Projeden ulusal ve uluslararası makale ve bildiriler üretileceği bildirilmiştir. Bu açıdan, projenin bilimsel ve akademik çıktılarına ulaşabilme potansiyeli yüksektir. Myoelektrik protez kullanan transradial amputelerin motor imgeleme yeteneğinin geliştirilmesine yönelik tedavi programlarının geliştirilmesinin ekonomik/ ticari/ sosyal çıktılara sınırlı ölçüde katkı sağlayacağı değerlendirilmiştir.

5. PROJE İLE İLGİLİ DİĞER GÖRÜŞLER

Projenin yürürlüğe alınması için sözleşme öncesi tamamlanması gereken belgeler verilen süre içinde (daha sonra e-posta ile bilgilendirme yapılacaktır) tamamlanmalıdır. Talep edilen belgelerin verilen süre içinde Kurumumuza iletilmemesi halinde destek kararı iptal edilecek ve sözleşme yapılmayacaktır.

- Proje ekibinde yer alan kişiler ile projenin yürütüleceği kurum/kuruluş arasında, projenin yürütülmesi esnasında veya sonucunda bir fikri ürünün ortaya çıkması durumunda bu fikri ürün üzerinde hak sahibi olacak olan gerçek/tüzel kişi(ler)in belirlenmesi amacıyla **Fikri Mülkiyet Hak Sahipliği Protokolü**'nün düzenlenmesi ve söz konusu Protokol'de ilgili kişilerin ıslak imzalarının bulunması gerekmektedir.

- Proje kapsamında gerçekleştirilecek çalışmalar için **Klinik Araştırmalar Etik Kurul Onay Belgesi** alınması

gerekmektedir.

- Makine ve Teçhizat Giderleri ile Hizmet Alımları bütçe kalemleri için **proforma fatura** sunulması gerekmektedir.

6. PROJENİN BÜTÇESİ VE GEREKÇESİNİN UYGUNLUĞUNA İLİŞKİN GÖRÜŞ VE ÖNERİLER

Proje bütçesi TÜBİTAK destek mevzuatı ve uygulamaları çerçevesinde düzenlenmesi kaydıyla sunulduğu şekli ile uygun bulunmuştur. Uygun bulunan bütçe aşağıda verilmiştir.

7. PROJE SÜRESİNİN UYGUNLUĞUNA İLİŞKİN GÖRÜŞ VE ÖNERİLER

Proje süresi, önerildiği haliyle 12 ay olarak uygun bulunmuştur.
